# On the Origin of the Red-Shifted Flavin Absorption Spectra in Fatty Acid Photodecarboxylase

**DOI:** 10.1021/acs.jpcb.5c05331

**Published:** 2025-12-18

**Authors:** Matteo Farina, Gianluca Dell’Orletta, Enrico Bodo, Isabella Daidone

**Affiliations:** † Department of Chemistry, University of Rome “La Sapienza”, Piazzale Aldo Moro 5, Rome 00185, Italy; ‡ Department of Physical and Chemical Sciences, 201850 University of L’Aquila, via Vetoio (Coppito 1), 67100 L’Aquila, Italy

## Abstract

Fatty acid photodecarboxylase (FAP) is one of the few known natural photoenzymes and has attracted considerable interest due to its ability to convert fatty acids into hydrocarbons upon photoexcitation of its oxidized flavin adenine dinucleotide (FAD) cofactor. Notably, FAD in FAP exhibits an absorption spectrum red-shifted by approximately 10–15 nm compared to many other flavoproteins. This shift might arise from the specific electrostatics of the binding pocket and/or the slightly bent conformation of the FAD, as suggested by the crystallographic data. During the photocycle, an even more red-shifted intermediate (FAD_RS_) has been observed, which ultimately reverts to the original state. In this work, we simulate the absorption spectrum of FAD inside FAP using a hybrid computational approach that combines quantum mechanics (QM) and molecular dynamics (MD) simulations in the Perturbed Matrix Method (PMM) framework. The computed absorption spectrum matches and explains the experimental one, not only validating the effectiveness of the MD-PMM approach but also revealing that the observed red shift primarily originates from the electrostatic environment provided by the protein matrix, whereas the effect of bending is comparatively minor. Additionally, we show that the formation of FAD_*RS*_ is unrelated to changes in active-site residue protonation or FAD conformation, but instead is likely to arise from a stable interaction between the flavin ring and bicarbonate, one of the proposed reaction products.

## Graphical abstract

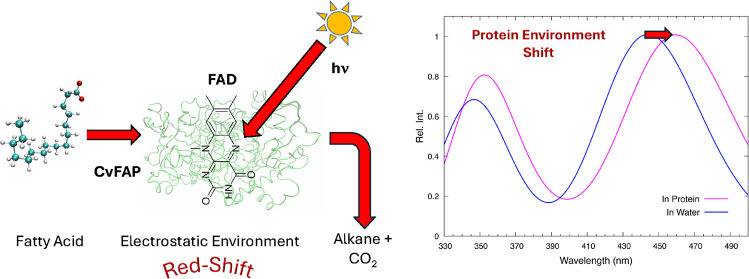

## Introduction

Flavins are heterocyclic compounds derived from riboflavin (vitamin B_2_) that play essential roles in diverse biological processes, including electron transfer, DNA repair, magnetoreception, and circadian rhythm regulation.^1–4^ When incorporated into proteins as prosthetic groups, flavins endow flavoproteins with versatile redox properties and the ability to mediate electron transfer processes with high specificity and efficiency.^5,6^ In addition to their roles in redox biochemistry, flavins exhibit strong fluorescence and participate in various light-dependent biological functions, including photoreception and light-induced signaling. A prominent example of such a system is fatty acid photodecarboxylase (FAP), a recently discovered flavoprotein that harnesses the excited state of its flavin adenine dinucleotide (FAD) cofactor to drive the oxidative decarboxylation of fatty acids. Upon exposure to blue light, FAP efficiently converts fatty acids into hydrocarbons,^7,8^ a reaction with significant potential for industrial purposes.^9,10^ FAP represents a rare case in which a flavin cofactor serves as the direct photochemical driver of catalysis, initiating substrate-centered radical formation from its excited state with quantum yields exceeding 80%.

Despite its technological potential and unique photochemical properties, the molecular details of FAP’s catalytic mechanism remain only partially understood. The crystal structure of Chlorella variabilis FAP (CvFAP) has recently been resolved, and its spectroscopic properties have been characterized in details.^7,8,11^ Notably, the absorption spectrum of the oxidized FAD cofactor in CvFAP, both in the absence of substrate (referred to as Apo) and in the presence of its fatty acid substrate (referred to as Holo), exhibits a significant red shift of approximately 10–15 nm compared with typical flavoproteins.^12,13^ Specifically, the position of the main absorption peak is red-shifted by 23 nm^7,14^ compared to lumiflavin in water (Figure 1 and Table 1).

**Figure 1:**
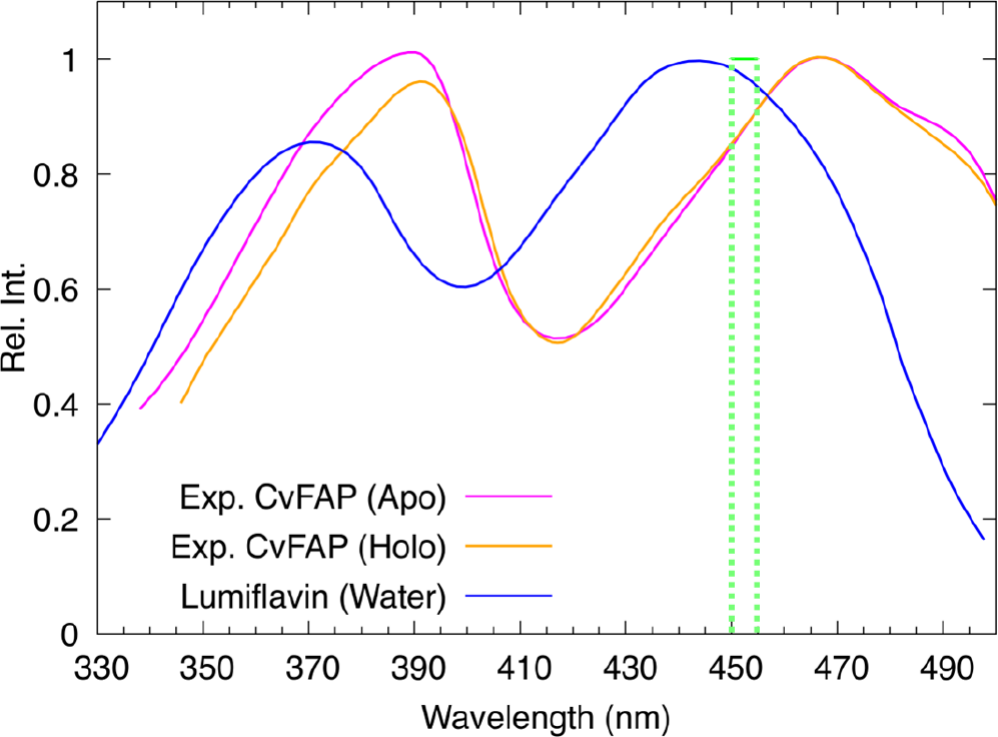
Comparison between the experimental UV–vis spectrum of lumiflavin in water (blue)^14^ and the spectra of FAD within the CvFAP.^8,15^ In CvFAP, both in the absence (Apo state, magenta) and in the presence (Holo state, orange) of the substrate, the main absorption peaks are red-shifted relative to lumiflavin in water, and there is a significant change in their relative intensity. It can be observed that the presence of the substrate has a moderate effect on the spectrum. The green box indicates the spectral range (450 to 455 nm) in which the first FAD absorption peak is typically observed in other flavoproteins.

**Table 1:** Transition Energies and Relative Intensity of the Experimental Absorption Peaks of FAD within CvFAP (Apo^8^ and Holo^15^) and Lumiflavin in Water^14^

reference	λ_1_ (nm)	λ_2_ (nm)	*I*_rel_
CvFAP Apo^8^	466	389	1.01
CvFAP Holo^15^	466	391	0.96
in water^14^	443	370	0.86

Analysis of the crystal structures has revealed that the oxidized FAD cofactor in CvFAP adopts a distinctive “butterfly-bent” conformation, defined by a *C*_4_–*N*_5_–*N*_10_–*C*_9_ dihedral angle ranging from 11.7 to 17.4°, depending on the temperature and dose.^7,11^ While bending of the flavin ring has been previously observed in reduced states, it is uncommon in the oxidized form and has been proposed as a contributing factor to the observed spectral shift.^7,8^ However, the electrostatic and hydrogen-bonding environments of the active site are also expected to significantly influence the electronic properties of the flavin chromophore. Despite these insights, the current literature lacks a quantitative distinction between the respective contributions of FAD bending and environmental perturbations to the observed spectral red shift. Adding to the complexity, previous studies^7,15^ have reported that, following photoinduced electron transfer from FAD to the substrate and subsequent reoxidation, a flavin species with an even more pronounced red-shifted absorption (approximately 10 nm further^7^), termed *FAD*_*RS*_, is formed. The mechanistic origin of this additional shift remains unclear. Possible contributing factors include a redistribution of electrostatic charges within the active site, a further increase in flavin bending, or the formation of new hydrogen bonds that stabilize specific excited-state conformations.

In this context, a deeper understanding of the absorption spectra and electronic properties of flavins within the CvFAP environment is essential. Accurate computational modeling, capable of accounting for environmental effects, such as hydrogen bonding, electrostatics, and protein dynamics, is crucial to interpret experimental observations and to shed light on the red shifts. To this end, we employ a hybrid quantum/classical strategy combining molecular dynamics (MD) simulations with the Perturbed Matrix Method (PMM)^16^ and Quantum Mechanics/Molecular Mechanics (QM/MM) calculations.^17^ The MD–PMM approach enables extensive sampling of the conformational space of the biosystem while incorporating the perturbative effects of the environment on quantum-mechanical observables. This allows the explicit inclusion of dynamic electrostatic and structural fluctuations.^18–25^ The method has previously been successfully applied to simulate the absorption spectrum of flavins in different environments, such as riboflavin in aqueous solution^20^ and in the riboflavin-binding protein,^21^ yielding results in excellent agreement with experimental data.

## Theoretical Methods

### Choice of the DFT Level of Theory

A benchmark of density functional theory (DFT) functionals was performed on lumiflavin at the QM/MM-optimized structure, which was obtained from the crystal structure, PDB entry 6YRZ^7^ (Figure 2). Details of the QM/MM optimization are provided in the dedicated section. Seven common DFT functionals were tested in QM/MM calculations of lumiflavin (the QM center) in CvFAP to compare the computed and experimental absorption spectra. The results are presented in Figure 3, where we report the mean absolute error of the peak energies between the computed and the experimental spectra of CvFAP. Both M06 and B3LYP provide the best agreement with experimental excitation energy, a result that is consistent with previous benchmarks reported in the literature.^2,26,27^ Although expected to provide an improvement over the hybrid functional, double-hybrid functionals were excluded from the benchmark because, in the current implementation of ORCA, it is not possible to compute the excited-state dipole moments required for PMM calculations and are thus unusable. As detailed in the Supporting Information (SI), Figure S1, both M06 and B3LYP functionals were also used in a preliminary MD-PMM calculation on a 100 ns-long molecular dynamics trajectory of lumiflavin in water. The spectra obtained using B3LYP showed better agreement with the experimental data, supporting its use in subsequent MD-PMM calculations.

**Figure 2:**
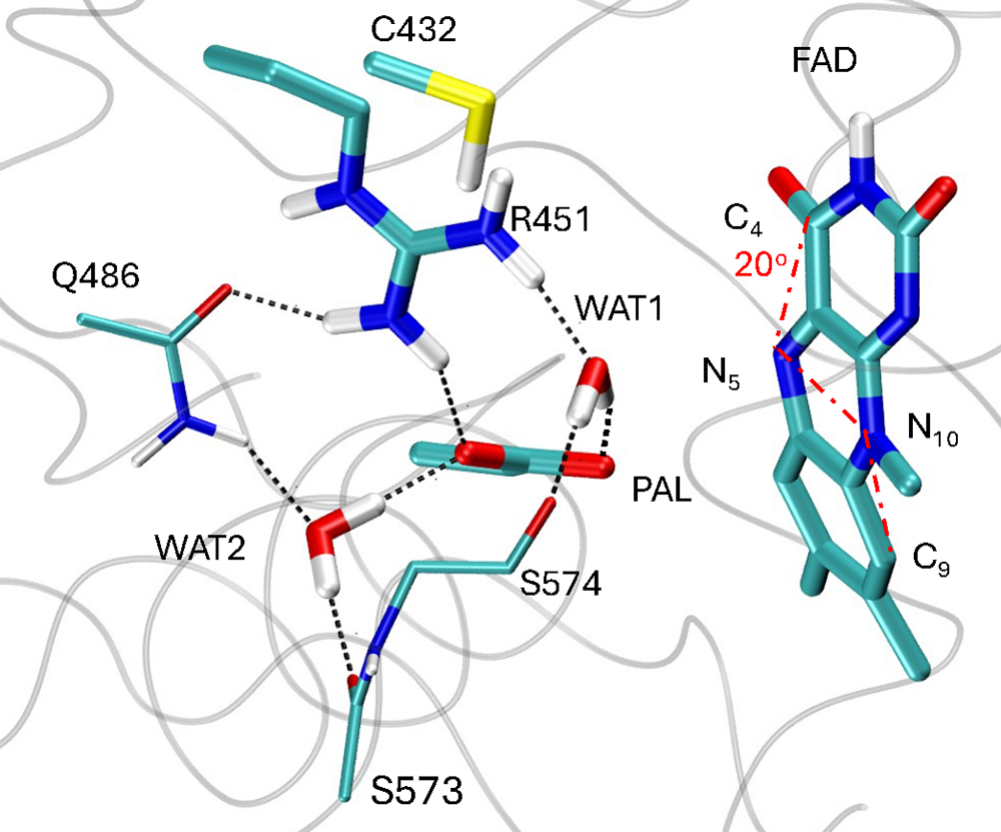
Active site of CvFAP in the QM/MM-optimized structure in the resting state. All atoms represented in the figure with thick lines are included in the QM part, while the backbone of S573 and of S574 and the side chain of Q486 (thin lines) are included in the MM and reported here to provide a better view of the active site. In this geometry, lumiflavin adopts a markedly bent conformation, with a C_4_–N_5_–N_10_–C_9_ dihedral angle (*d*_1_) of approximately 20°, about 3° greater than in the initial crystal structure at 100 K. The *d*_1_ angle is highlighted with red dashed line.

**Figure 3:**
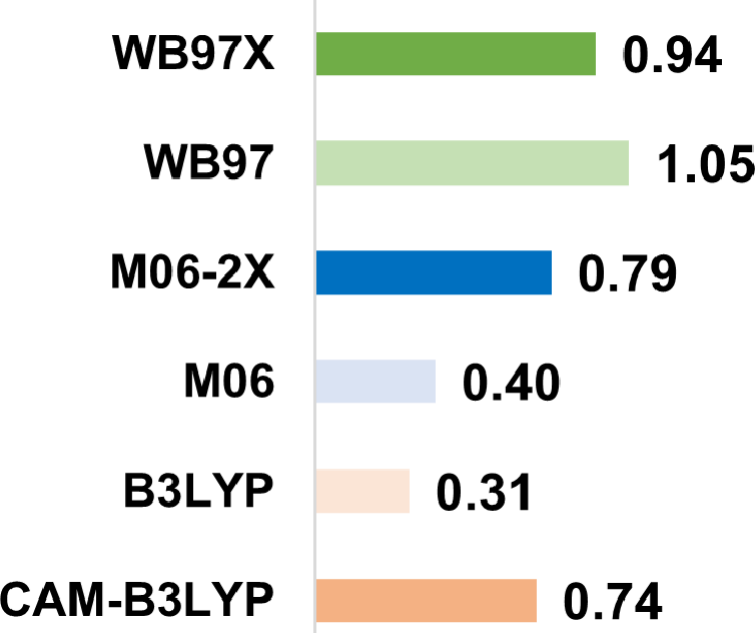
Mean absolute error (MAE) on peak energies (in eV) between the QM/MM and the experimental spectra of CvFAP for each DFT functional (basis set def2-TZVP). The QM region includes the lumiflavin moiety of FAD, while the environment is modeled at the MM level.

### Molecular Dynamics Simulations

All MD simulations were performed using the GROMACS software^28^ with the CHARMM36 force field.^29,30^ For the simulation of FAD in Apo CvFAP, the protein–cofactor complex, taken from the crystallographic structure (PDB ID:6YRZ), was placed in a periodic dodecahedral box with side lengths of 10.3 nm and solvated in water using the TIP3P model.^31^ Counterions (Na^+^) were added to neutralize the total system charge. Long-range Coulombic interactions were treated using the Particle Mesh Ewald (PME) method,^32^ with a Fourier spacing of 0.132 nm and a real-space cutoff of 1.2 nm. The Lennard–Jones interactions were also truncated at 1.2 nm. A time step of 0.001 ps was used, and bond lengths involving hydrogen atoms were constrained using the LINCS algorithm.^33^ During the minimization, heating, and equilibration steps, the heavy atoms of the protein, the C_α_ atoms, and the heavy atoms of the FAD cofactor were restrained to their initial positions using a force constant of 1000 kJ mol^–1^ nm^–2^. During the heating phase, the temperature was increased from 50 to 300 K over 100 ps. An MD simulation was conducted for 100 ns. The system was initially equilibrated in the NVT ensemble (constant number of particles, volume, and temperature) at 300 K for 2 ns using the Berendsen thermostat,^34^ with a relaxation time constant of 0.5 ps. This was followed by a 1 ns equilibration in the NPT ensemble (constant number of particles, pressure, and temperature), also using the Berendsen thermostat (relaxation time constant of 0.5 ps) and the Berendsen barostat^34^ with a relaxation time constant of 0.5 ps and a compressibility of 4.5 × 10^–5^ bar^–1^ at 300 K and 1 bar. After equilibration, a 100 ns production MD simulation was performed in the NPT ensemble at 300 K and 1 bar by using a 2 fs time step. Temperature and pressure were controlled using the Nosé–Hoover thermostat,^35^ with a relaxation time constant of 1.0 ps, and the Parrinello–Rahman barostat,^36^ with a relaxation time constant of 5.0 ps and a compressibility of 4.5 × 10^–5^ bar^–1^.

For the 100 ns-long MD simulation in water, the same simulation protocol was used (i.e., simulation parameters, force field, equilibration, and so forth). A planar molecule of lumiflavin was centered in a cubic box (side length = 4 nm) and solvated with 1517 water molecules.

#### Essential Dynamics Analysis

Quantitative characterization of the intramolecular conformational changes in lumiflavin relied on principal component analysis (PCA) of the covariance matrix of the positional fluctuations of the carbon and nitrogen atoms of the isoalloxazine moiety, as described elsewhere.^37^ This matrix was built from the equilibrated portion of the Apo CvFAP trajectory (beyond 10 ns), and its diagonalization yielded the principal directions of the large-amplitude concerted motions (essential eigenvectors) that characterize the essential subspace of lumiflavin internal dynamics. The first eigenvector largely corresponded to the butterfly bending mode (see the Results section).

### DFT Calculations on the Isolated Lumiflavin

Starting from representative structures extracted from the MD simulation (see the Results section), gas-phase optimizations of the lumiflavin moiety of FAD were carried out at the B3LYP/def2-TZVP level of theory. Geometric constraints were applied to preserve configurations close to those observed in the MD simulations. In particular, six dihedral angles associated with the characteristic “butterfly bending” motion (C_4_–N_5_–N_10_–C_9_, C_6_–N_5_–N_10_–N_1_, C_7_–C_6_–C_5a_–N_5_, C_8_–C_9_–C_9a_–N_10_, N_10_–C_10a_–N_1_–C_2_, and N_5_–C_4a_–C_4_–N_3_) were constrained during the optimizations. Time-dependent DFT (TD-DFT) calculations were carried out on the optimized structures using the B3LYP functional and the def2-TZVP basis set^38^ to obtain gas-phase spectra. TD-DFT was also used to compute the unperturbed ground-state energy, the excitation energies of the first 10 excited states, and both ground-to-excited and excited-state dipole moments (reported in Tables S1 and S3 of the SI), which serve as input for the MD-PMM framework. Excited-to-excited-state transition dipole moments were neglected, in agreement with previous studies,^20,39^ showing that their contribution to the absorption spectrum is negligible.

For the calculations in aqueous solution, only the planar FAD structure was considered, as molecular dynamics simulations indicated that lumiflavin predominantly adopts a planar conformation in water.

All calculations were carried out using ORCA version 6.0.1.^40,41^

### Perturbed Matrix Method Calculations

The main theoretical features of the MD–PMM^16,18,42^ and its application for calculating absorption spectra have already been described in previous works;^19–21^ therefore, only the essential aspects are reported here. The MD–PMM, like other hybrid computational methods, is based on partitioning the system into a quantum center (QC), in the specific case lumiflavin, treated at the electronic level and the rest of the system modeled as an atomistic, semiclassical perturbing subsystem interacting with the QC. In the first step, an MD simulation of the entire system was carried out for 100 ns (see section Molecular Dynamics Simulations). In the second phase, DFT calculations (with the setup described in the previous section) were used to determine a set of unperturbed electronic eigenstates Φ_*j*_^0^, which are eigenfunctions of the QC unperturbed electronic Hamiltonian matrix *Ĥ*_0_. The QC perturbed electronic Hamiltonian matrix *Ĥ* is built according to eqs 1 and 2:

$$\hat{H}={\hat{H}}_{0}+\hat{I}qV+\hat{Z}+\Delta V$$ 1

$$\hat{Z}=-E\langle \Phi_{j}^{0}|\hat{\mu }|\Phi_{j\prime }^{0}\rangle$$ 2

where *q* is the total QC charge, **μ̂** is the dipole operator, and *V* and **E** are, respectively, the electrostatic potential and field that the environment exerts on the QC center of mass at each frame of the MD simulation. Δ*V* includes all the other terms treated as a simple short-range potential that is not included in the current calculations, and *Î* is the identity matrix. In the MD-PMM approach, the Born–Oppenheimer approximation is considered true, thus implying that the electronic eigenfunctions are independent of the nuclei motion. Each Φ_*j*_^0^ depends only on the internal nuclear coordinates of the QC and is invariant for the QC roto-translations. In this specific case, each Φ_*j*_^0^ is defined by the QC conformational coordinates, while all the other internal coordinates are energy-minimized at each position of the conformational ones. At each MD frame, the perturbed electronic Hamiltonian matrix is constructed and diagonalized. This procedure yields a trajectory of perturbed eigenvalues and eigenvectors, which are used to compute the quantum observables of interest. In particular, for each MD frame, perturbed transition frequencies (ν) and transition dipoles (**μ**_**i** **j**_) are obtained.

For the calculation of the absorption spectrum, the ν and **μ**_**i** **j**_ distributions were evaluated using an appropriate number of intervals in the frequency space. These distributions are then used to calculate the molar extinction coefficients for the ground-to-*n*th excited-state transitions (ε_0,*n*_), from which the absorption spectrum is derived according to eqs 3 and 4:

$$\varepsilon_{0,n}(\nu )=\underset{\nu_{\mathrm{ref}}}{\sum }\frac{T_{A}(\nu_{\mathrm{ref}})\eta (\nu_{\mathrm{ref}})h\nu }{N}\frac{e^{\left[-(\nu -\nu_{\mathrm{ref}}{)}^{2}/(2\sigma^{2})\right]}}{\sigma \sqrt{2\pi }}$$ 3

$$T_{A}(\nu_{\mathrm{ref}})=\frac{|\mu_{0,n}(\nu_{\mathrm{ref}}){|}^{2}}{6\epsilon_{0}c\hbar^{2}}$$ 4

Here, ν_ref_ is the frequency at the center of each interval, η­(ν_ref_) is the number of corresponding MD frames, and |μ_0,*n*_|_ν_ref__^2^ is the mean transition-dipole squared norm in the interval. *ℏ* = *h*/2π, where *h* is the Planck constant, ϵ_0_ is the dielectric constant in vacuum, *c* is the speed of light, and σ^2^ is the variance produced by the neglected semiclassical vibrations (σ = 0.001). Consistent with the MD–PMM approach,^20^ a scaling factor of 0.94 was applied to all calculated energies in the spectra. This factor was chosen to ensure good agreement between the position of the first experimental absorption peak of lumiflavin in water and its theoretical counterpart. However, the scaling is applied purely as a multiplicative factor to the energy values (in eV) and does not affect the relative intensities of the absorption signals. After applying the scaling factor to both unperturbed and perturbed energies, the values are converted to wavelengths (in nm).

### Quantum Mechanics/Molecular Mechanics Calculations

QM/MM calculations were employed to optimize and characterize the corresponding electronic excited states, two structures: one representative of the reactant state (containing the substrate molecule in the active site) and another representative of the product state (containing the alkane product and a bicarbonate anion).

The initial structure of the system was taken from the crystal structure of CvFAP at pH 8.5 (PDB entry 6YRZ).^7^ A molecule of palmitate was added to replace the cocrystallized substrate, i.e., stearic acid. The protonation state of all ionizable residues and of the substrate was assigned according to previous experimental studies conducted at the same pH.^7,11,43^ To reproduce an experimental pH of 8.5, residue protonation states were assigned using PROPKA.^44^ The protonation state of histidine residues was assigned via visual inspection, considering potential hydrogen bonding and the local chemical environment. The structure was minimized using the AMBER force field,^45–47^ constraining the positions of all heavy atoms, with the MM module of ORCA 6.0.^40,41^ Minimization was followed by QM/MM optimization, which was carried out using the electrostatic embedding scheme^48^ implemented in ORCA 6.0 For the boundary region, the charge-shifting scheme was employed, and hydrogen link atoms were used to saturate the valency of atoms in the QM region.^17,49,50^ For the calculation on the reactant structure, the high-level QM region included the side chains of Arg451 and Cys432, the headgroup of palmitate (the −CH_2_CH_2_CO_2_^–^ fragment), the lumiflavin moiety of FAD, and two water molecules hydrogen-bonded to the palmitate (Wat1 and Wat2). For the product structure, the heads of palmitate and Wat1 were replaced by a bicarbonate ion and the head of pentadecane (the −CH_2_CH_3_ fragment).

In all the calculations, the QM region was treated at the DFT level using the B3LYP functional^51,52^ with the def2-TZVP basis set,^53^ employing the RIJCOSX approximation^54^ along with an appropriate auxiliary basis set to speed up the calculations. The MM part, modeled with the AMBER force field, was divided into two regions: an active region, where atoms were allowed to move during optimization, and a static region, which remained constrained and acted solely as an electrostatic environment. A large shell of approximately 350 atoms near the QM region was included in the active MM region. This included residues Ile130, Phe134, Ala171, Leu173, Cys432, Arg451, Val463, Tyr466, Gln486, Ser573, Ser574, Asn575, and Gly622 and the remaining portions of FAD and palmitate, while the rest of the system was considered as the static MM part. Optimization was performed using the BFGS update method^55^ with default convergence criteria: an energy change of 5 × 10^–6^ E_h_, a maximum gradient component of 3 × 10^–4^ E_h_/bohr, an RMS gradient of 1 × 10^–4^ E_h_/bohr, a maximum step component of 4 × 10^–3^ bohr, and an RMS step tolerance of 2 × 10^–3^ bohr. Crystallographic water molecules were included in the static MM part, but the remaining solvent was not considered. In the case of the product state, starting from the optimized crystallographic structure and maintaining the same selection for the QM region, the heads of palmitate and Wat1 were replaced by a bicarbonate ion and the heads of pentadecane. The bicarbonate anion was placed in close contact to the FAD moiety according to the configuration reported in the work by Sorigué et al.^7^ Specifically, we reproduced the product structure corresponding to Pathway II. The resulting structure was optimized at the B3LYP/def2-TZVP level within the QM/MM framework. On both the optimized reactant and product structures, TD-DFT calculations were performed at the same level of theory, without the TDA approximation and considering the first 20 excited states.

## Results and Discussion

### Gas-Phase UV–Vis Absorption Spectra: The Effect of Lumiflavin Bending

To disentangle the effect of molecular bending from that of electrostatic perturbations by the environment on the red-shifting of the UV–vis absorption spectrum of lumiflavin within CvFAP, we first focused on the influence of intramolecular conformational changes. Specifically, we evaluated in the gas phase how the characteristic butterfly bending impacts the UV–vis peak positions. To this end, we analyzed the butterfly motion along the MD trajectory of CvFAP to identify a collective variable describing this bending and to extract representative conformations covering its full range.

A principal component analysis (PCA) was performed on the heavy atoms of the lumiflavin ring system along the MD trajectory to characterize its collective motions (see the Methods section for details). The first principal component corresponded to the typical butterfly bending mode, as illustrated by five representative structures extracted along this mode, from one extreme to the other (Figure 4, panel A). From the MD trajectory, five configurations were selected to span the range of projections along this first PCA mode (Figure 4, panel B), corresponding to variations of the key inter-ring dihedral angle, *d*_1_, between 0° and 20° (Table 2). For each structure, the QM region (the lumiflavin moiety) was then isolated and relaxed, and gas-phase excited-state energy calculations were performed (see Methods section). Analysis of the energy of the first and second excited states, reported as a function of the projection along the first PCA eigenvector given in Table 2, revealed only minor variations. The resulting spectra (Figure 5 and Table 2) indicate that bending induces only a slight red shift in both absorption peaks, with a maximum shift of about 4 nm when comparing the most bent conformation (B5, *d*_1_ = 20°) to the planar geometry (B1, *d*_1_ = 0°). Regarding the corresponding intensities, the first excited state remains nearly unchanged, whereas the intensity of the second peak increases progressively from the planar to the bent structure.

**Figure 4:**
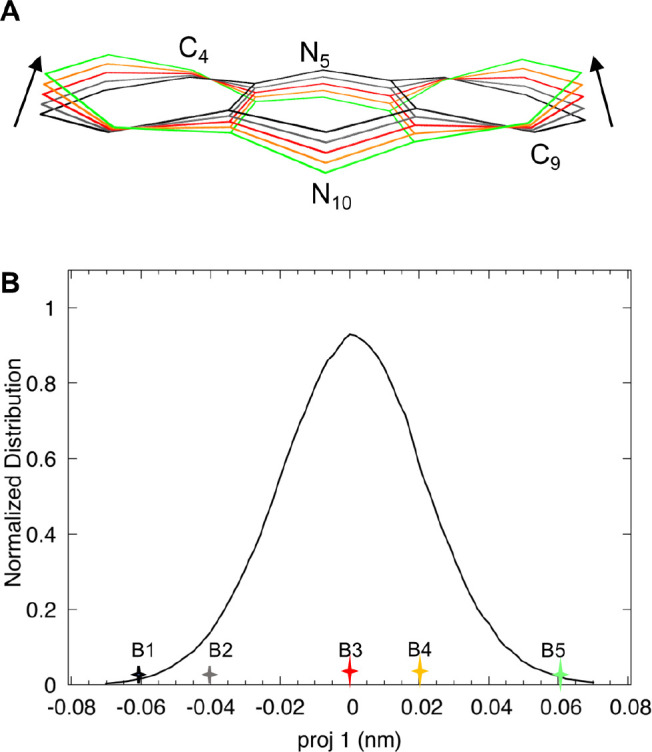
(A) Representative structures extracted along the first essential eigenvector corresponding to the butterfly bending mode obtained by the principal component analysis of the heavy-atom coordinates of the lumiflavin ring system along the MD trajectory of Apo CvFAP. The arrow indicates the direction of the projection along the first eigenvector, from negative to positive values. (B) Distribution of the projection of the MD trajectory on the first eigenvector (proj 1).

**Table 2:** Projection along the First Essential Eigenvector (proj 1), Bending Angle (*d*_1_), First (λ_1_) and Second (λ) Excited-State Energies, and Their Relative Intensity I_rel_, for the Five Representative Structures Extracted from the MD Trajectory of Apo CvFAP

	proj 1	*d*_1_	λ_1_ (nm)	λ_2_ (nm)	*I*_rel_
B1	–0.06	0°	436.2	342.8	0.76
B2	–0.04	3°	436.7	344.0	0.83
B3	0.00	10°	437.2	343.6	0.83
B4	0.02	18°	439.2	345.8	0.91
B5	0.06	20°	441.0	347.0	0.97

**Figure 5:**
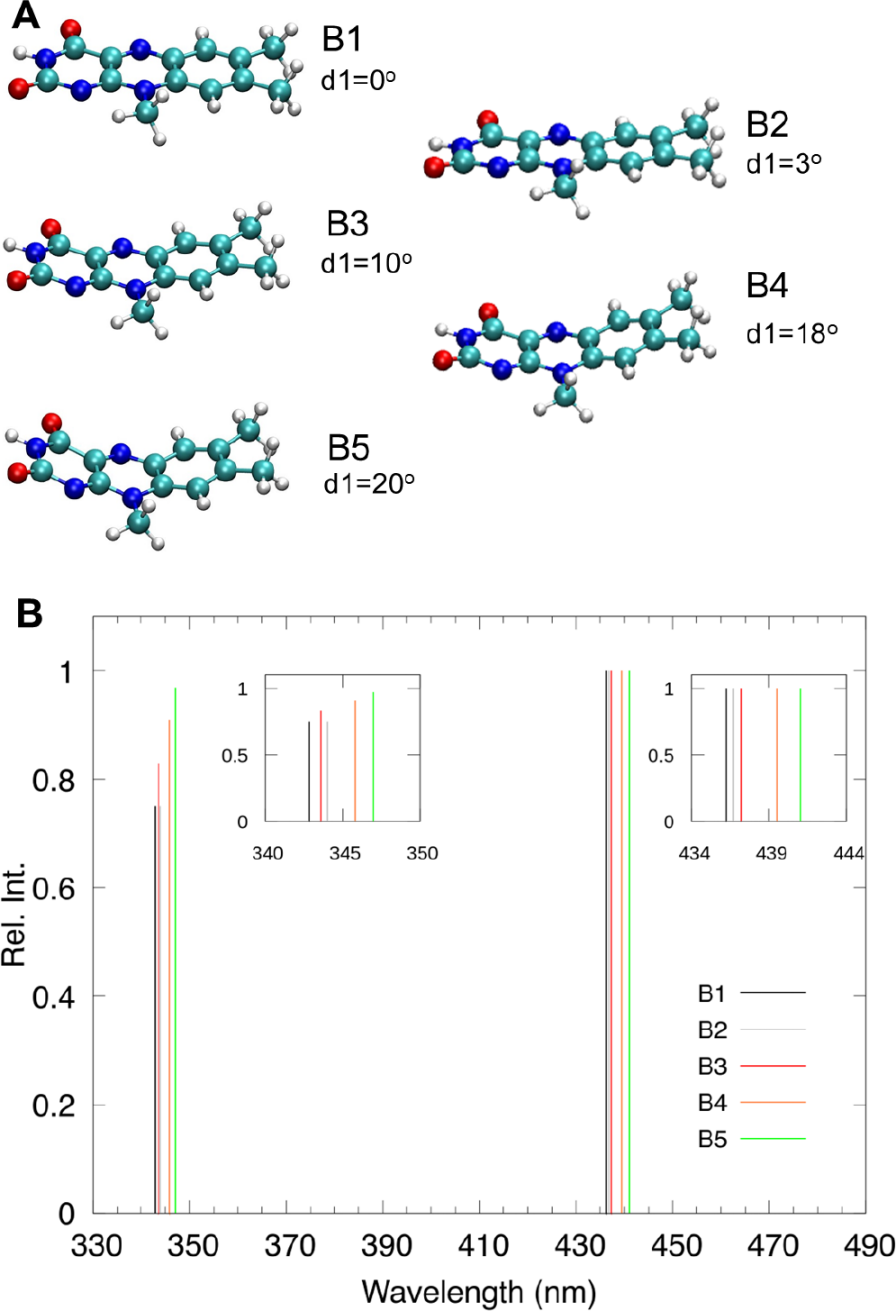
(A) Structures of five lumiflavin conformations extracted along the first essential eigenvector and optimized at the B3LYP/def2-TZVP level of theory, featuring five different degrees of bending of the C_4_–N_5_–N_10_–C_9_ dihedral angle (*d*_1_). (B) Energies and normalized intensities of the first two bright states, calculated in the gas phase using TD-DFT at the same level of theory, for the five conformers. A scaling factor of 0.94 was applied to the energies (in eV).

These results clearly demonstrate that bending has a modest effect on the electronic properties of lumiflavin. In particular, the magnitude of the bending-induced bathochromic shift is small, while the change in the relative intensity of the absorption peaks is slightly more pronounced.

### MD-PMM Calculations of the UV–Vis Absorption Spectrum in CvFAP

To calculate the absorption spectrum of FAD in the protein environment and in water for comparison purposes, we used the MD-PMM approach (see the Methods section). Two MD simulations were performed using the CHARMM force field implemented in GROMACS: one in explicit water and the other in the Apo form of CvFAP. Since the lumiflavin chromophore predominantly adopts a planar configuration in the water simulation, the unperturbed eigenstates of the planar structure B1 were used. For the simulation in Apo CvFAP, all frames from the MD trajectory were categorized into three groups based on the conformation of the lumiflavin chromophore: planar (27% of the frames), partially bent (44% of the frames), and bent (29% of the frames) (see note to Table 3 for the definition of these intervals). Each group was assigned the corresponding unperturbed reference geometry (B1, B3, and B5, respectively) and associated eigenstates for the PMM calculations. The individual spectra associated with the different bending angles are reported in Figure S2 of the SI, while the total spectrum is shown in Figure 6, panel A. Notably, for all three conformers, the inclusion of the electrostatic perturbation exerted by the environment induces a red shift of the main peak of approximately 10–15 nm compared to the corresponding gas-phase values. Moreover, the relative shift between the planar and 20° bent structures remains essentially unchanged from the gas-phase spectra, at around 4 nm.

**Figure 6:**
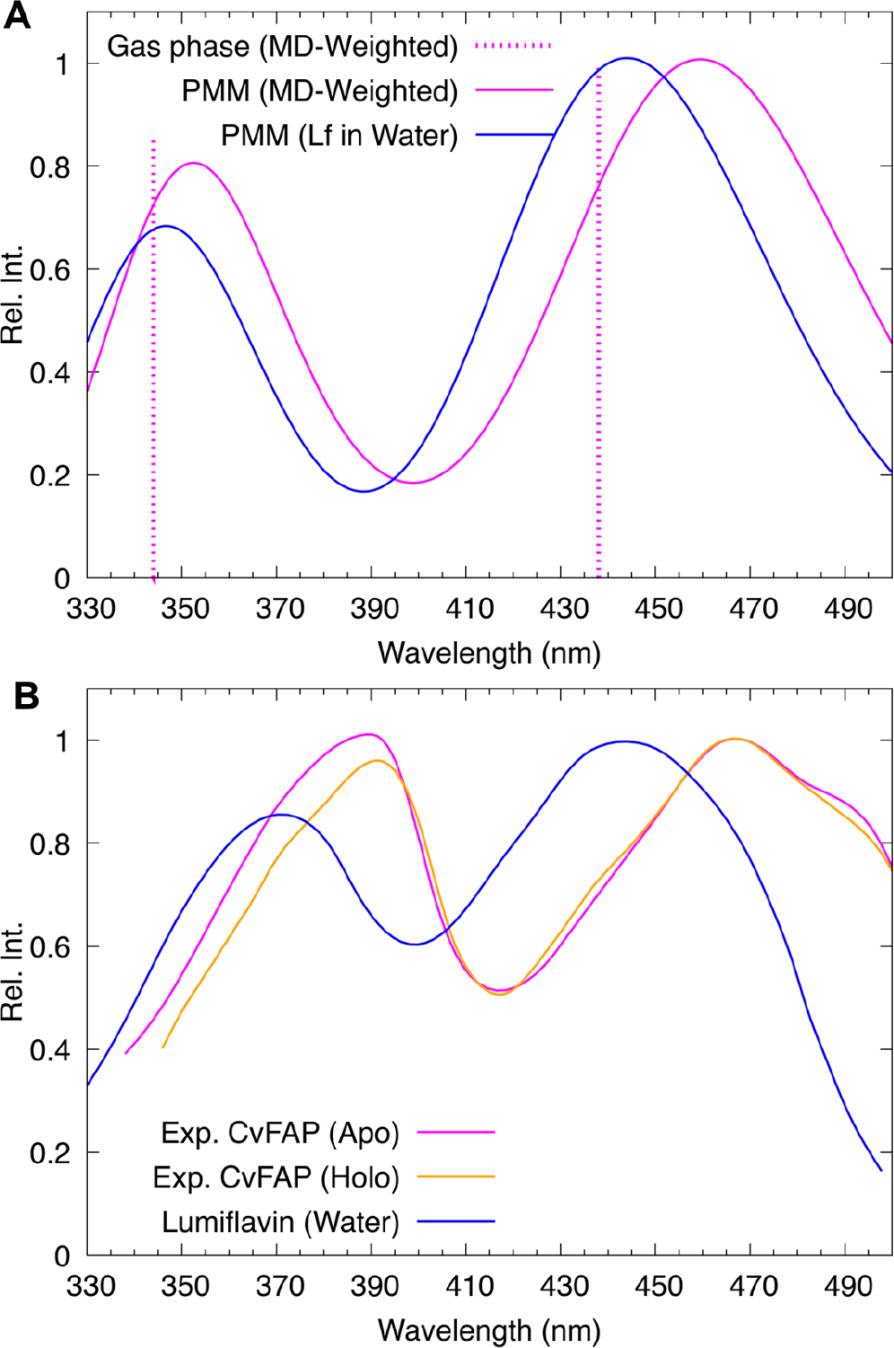
Comparison between MD-PMM-calculated (A) and the experimental (B) lumiflavin spectra in water and in CvFAP. The total MD-PMM spectrum in CvFAP weighted by the MD populations corresponding to the three different basins (represented by structures B1, B3, and B5) (magenta line) closely reproduces the experimental one, showing good agreement in the red shift of the first absorption peak relative to that in water (16 vs 23 nm). The energy of the first two peaks in the gas phase, also weighted by the MD populations of the three different basins, is shown as a dashed line for comparison, highlighting the substantial contribution of electrostatic perturbations to the observed red shift. A scaling factor of 0.94 was applied to the computed energies (in eV).

For what concerns the total absorption spectrum, it can be observed that the agreement with the experimental spectra (reported in panel B of Figure 6) is very good (see also Table 3 for the specific peak values). In particular, the shift of the first peak in CvFAP with respect to water is very close to the experimental one (16 versus 23 nm).

**Table 3:** Simulated Energies of the Main UV–Vis Absorption Peaks for Lumiflavin within CvFAP in Various Conformations and in Water Calculated with the MD-PMM Approach

system	λ_1_ (nm)	λ_2_ (nm)
MD-PMM computational data^a^		
planar	458	352
partially bent	458	351
bent	462	355
MD weighted	459	352
LF in water	443	346
gas-phase computational data^b^		
planar (gas phase)	436	342
MD weighted (gas phase)	438	344
experimental data^b^		
CvFAP Apo^8^	466	389
CvFAP Holo^15^	466	391
LF in water^14^	443	370

a ‘Planar’, ‘Partially bent’, and ‘Bent’ refer to calculations performed on three structural basins identified from the Apo CvFAP MD simulation, defined as follows: planar (*d*_1_ < 6°, 27% of sampled structures), partially bent (*d*_1_ 6°–12°, 44%), and bent (*d*_1_ > 12°, 29%). ‘MD weighted’ refers to values averaged over the MD simulation of Apo CvFAP according to the population of each basin. ‘LF in water’ indicates data of lumiflavin in water computed using the MD-PMM approach. ‘Planar (gas phase)’ and ‘MD weighted (gas phase)’ refer to the planar geometry and to the values averaged over the MD simulation of Apo CvFAP according to the population of each basin. Experimental reference values are taken from Wu et al. (2021, CvFAP Apo), Heyes et al. (2020, CvFAP Holo), and Sikorski et al. (2001, lumiflavin in water).

b Gas-phase values and experimental data are provided for comparison.

To quantify the individual contributions of bending and electrostatic perturbations, we analyzed the total energy shift between the first peak of the planar gas-phase spectrum and the corresponding peak in the protein environment, amounting to 23 nm (Table 3). Most of this red shift originates from protein and solvent effects, as shown by comparing the first absorption peak of the planar gas-phase structure (436 nm) with that of the MD population-weighted average value in the gas phase (438 nm) and in the protein environment from MD–PMM calculations (459 nm) (Table 3). The small 2 nm shift observed in the gas phase reflects intramolecular conformational effects, whereas the much larger 21 nm shift (from 438 to 459 nm) arises from electrostatic perturbations induced by the protein and solvent environment.

This result demonstrates that the protein environment plays a major role in the pronounced red shift observed in the UV–vis spectrum of FAD within CvFAP. It should be noted that the exact spectral shift between water and the CvFAP environment depends on the choice of the force field used in the MD simulations. However, the relative contributions of the bending angle and the environmental electrostatic perturbation to the red shift, with respect to water, are not expected to be significantly affected by the choice of the force field.

### Origin of the Red-Shifted Intermediate State in CvFAP

The catalytic cycle in CvFAP begins with a forward electron transfer from the bound fatty acid to the oxidized flavin, generating a flavin radical anion and a fatty acid radical. The latter undergoes decarboxylation, producing an alkyl radical and a CO_2_. The flavin radical is then reoxidized via back electron transfer within approximately 100 ns, donating the electron required to reduce the alkyl radical to the corresponding alkane. This reoxidation step leads to the transient red-shifted flavin state, FAD_*RS*_, which returns to the ground state within ≈4 ms.^7^ While a subpopulation of semiquinone flavins that are not reoxidized may contribute to the observed spectral shift (≈10 nm), it has been hypothesized to originate from changes in the charge distribution at the active site. According to Heyes et al., the red shift might arise primarily from a change in the protonation state of Cys432 from its neutral to its anionic form.^15^ Differently, according to Sorigué et al., the red shift is attributed to the negative charge of the deprotonated carboxylate being neutralized by the formation of CO_2_ and deprotonated Arg451.^7^

To investigate the role of the protonation states of Arg451 and Cys432, the absorption spectrum was calculated using the PMM approach, based on the same MD simulation of Apo CvFAP, but with modifications to the protonation states of the corresponding side chains. In one test, the charge of Arg451 was changed from +1 to 0, setting its side-chain charges according to the neutral state; in a second test, the side-chain partial charges of Cys432 were changed from 0 to −1, setting its side-chain charges according to the anionic side chain. This was achieved using the corresponding partial charges of the same force field. The resulting spectra, reported in Figure 7, show that both modifications lead to a blue shift of the absorption peaks, contrary to the expected red shift. The individual spectra arising from the three different conformational basins of lumiflavin are reported in Figure S3 of the SI.

**Figure 7:**
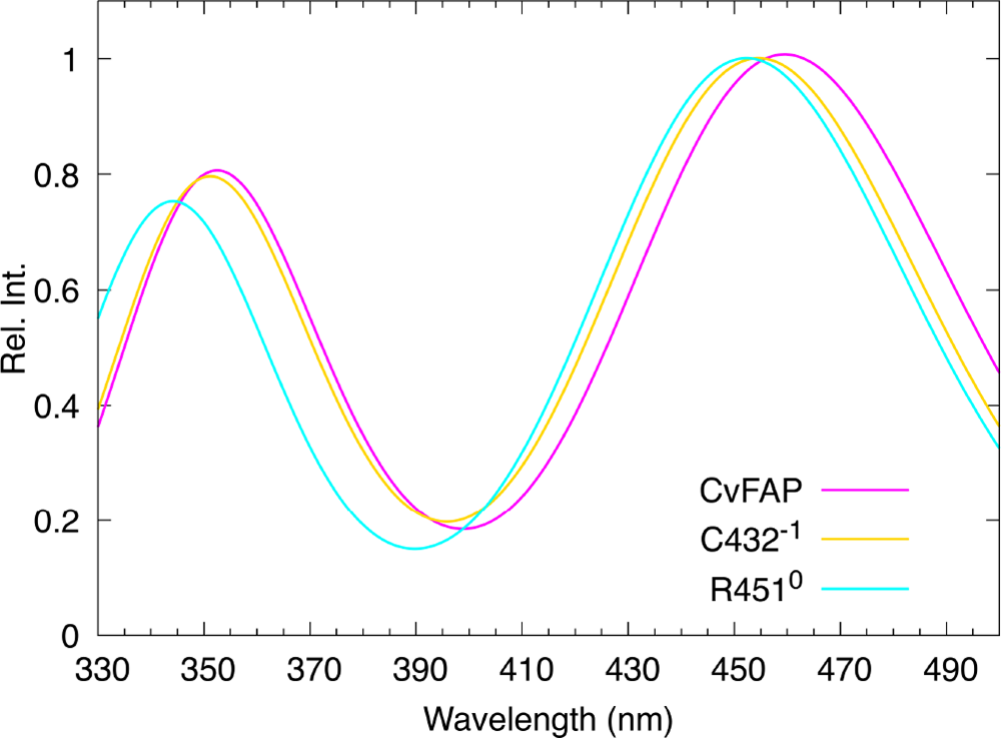
Comparison between the spectrum in CvFAP obtained using MD-PMM calculations with the standard Apo-state charges and those with altered charges, namely, Arg451 set to neutral (0) and Cys432 set to negatively charged (−1). In both cases, the charge alterations result in a blue shift, rather than in a red shift, which is slightly more pronounced for the neutral Arg451. The spectra are calculated as averaged over the MD simulation according to the population of the conformational basins of lumiflavin in the Apo CvFAP MD simulation (see Table 3 for details).

These results suggest that the observed red shift does not originate from changes in the charge of the active site due to the deprotonation of either Arg451 or Cys432. We therefore investigated the possibility that the red shift observed in the FAD_*RS*_ intermediate arises instead from a strong perturbation of the FAD electronic states caused by the formation of the products of the reaction. According to the reaction pathway identified as the most favorable in the foundational work of Sorigué et al.^7^ (referred to as Path II in their study), after the proton-coupled electron transfer event leading to alkane formation, during which residue Arg451 donates a proton and is subsequently reprotonated by Wat1, CO_2_ and the newly formed OH^–^ combine to yield a bicarbonate anion which establishes a strong interaction with the N5 atom of FAD. Because of the specific nature of this interaction, which may involve direct orbital overlap and charge delocalization between FAD and the bicarbonate anion (vide infra), the PMM approach, which is based on a perturbative treatment of electrostatic fluctuations, is not suitable to accurately capture its effect on the electronic properties of the FAD. We therefore employed QM/MM calculations, which explicitly describe the interaction at a full quantum-mechanical level. This analysis is intended to provide a qualitative interpretation of the origin of the red shift.

To this end, we performed QM/MM geometry optimizations of both the reactant structure (Figure 2) and the FAD_*RS*_ intermediate containing the bicarbonate anion, here referred to as the product structure (Figure 8). The corresponding absorption spectra were computed by using TD-DFT at the B3LYP/def2-TZVP level within a QM/MM framework. The QM region included the side chains of Cys432 and Arg451, the catalytic water molecules (Wat1 and Wat2), the lumiflavin moiety of FAD, and the headgroup of palmitate in the reactant structure. In the product structure, the heads of palmitate and Wat1 were replaced by a bicarbonate ion and the heads of pentadecane. The results, summarized in Figure 9, reveal a red shift of 23 nm in the absorption spectrum of the product relative to the reactant. This value is in qualitative agreement with the experimentally observed red shift of approximately 10 nm for the FAD_*RS*_ intermediate.^7,15^

**Figure 8:**
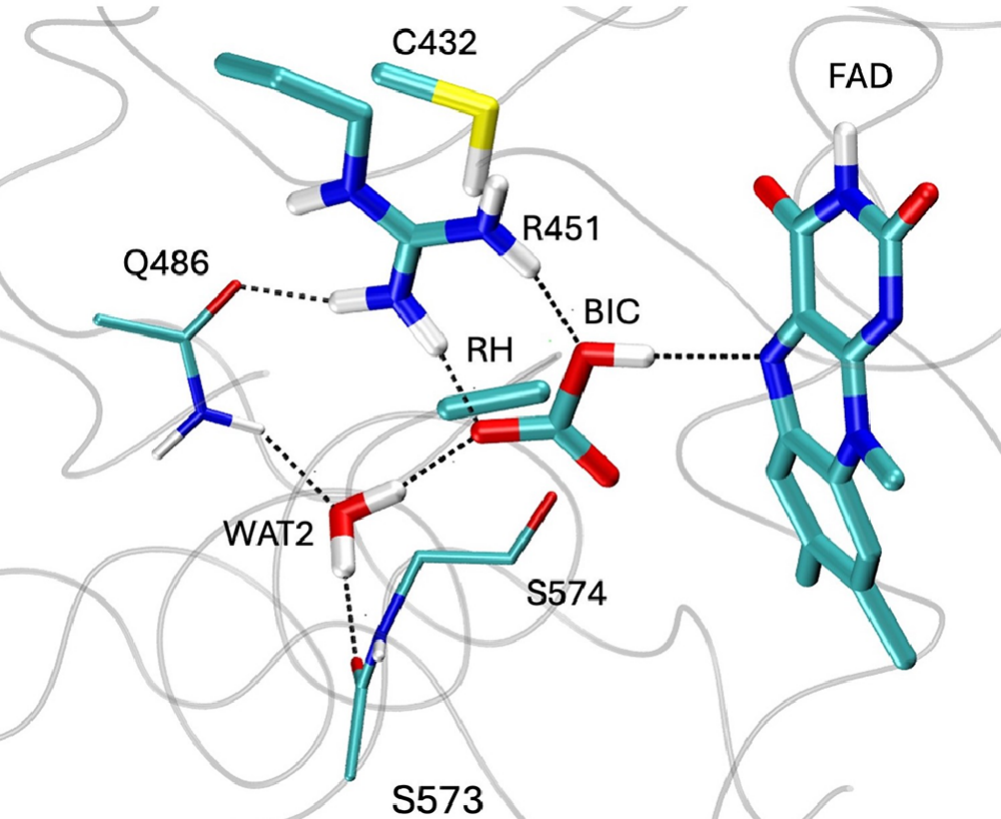
Optimized product structure at the B3LYP-def2-TZVP level of theory. All atoms represented in the figure with thick lines are included in the QM part, while the backbone of S573 and of S574 and the side chain of Q486 (thin lines) are included in the MM.

**Figure 9:**
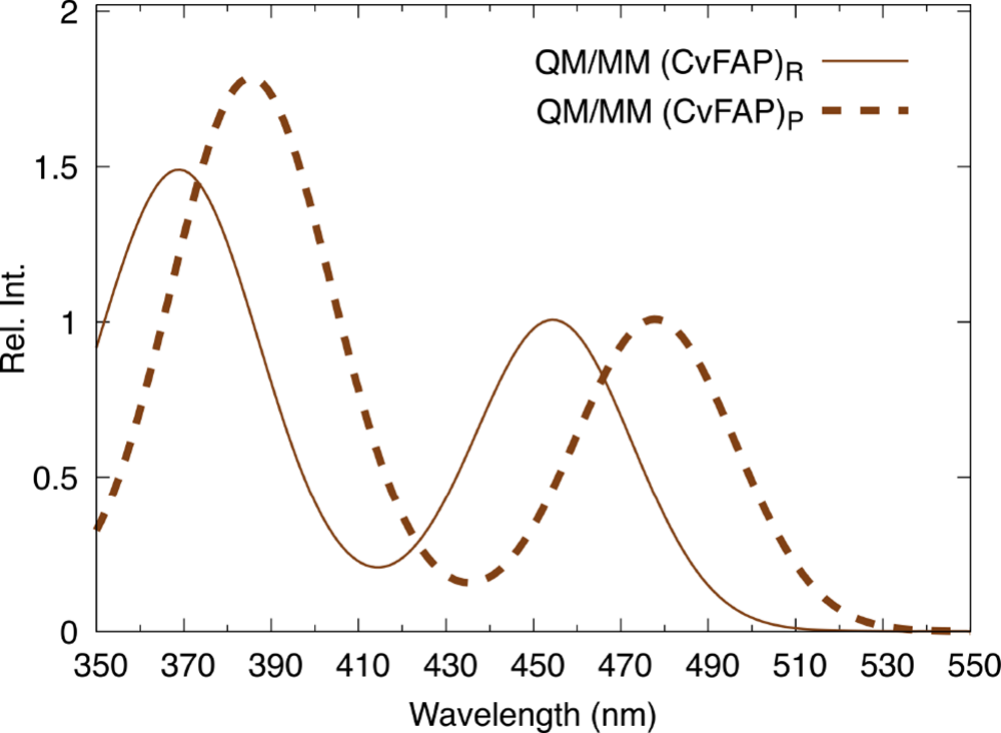
Spectra of the product (dashed line) and the reactant structure (solid line) of Holo CvFAP calculated at the QM/MM level. Our data are in qualitative agreement with experimental data, showing a red-shifted spectrum for the product structure.

Analysis of the optimized geometries showed no significant variation in the bending of the FAD isoalloxazine moiety, which remained close to 20° in both structures. However, the calculations revealed the presence of a strong hydrogen-bonding interaction between the flavin *N*_5_ atom and the hydroxyl group of the bicarbonate anion (O–N distance: 0.32 nm; N–O–H angle: 13.5°), which may contribute to the observed spectral shift. Kabir et al.^56^ demonstrated that the presence of a charged residue near the lumiflavin moiety of FMN can polarize the N_5_ or N_1_ atoms, resulting in noticeable shifts of the absorption peaks. In our case, however, Hirshfeld charge analysis did not reveal any change in the partial charges of N_1_, N_5_, and C_4a_ when comparing the reactant and product structures. These values remained nearly identical in both states, excluding a major electrostatic contribution arising from the hydrogen bond.

Therefore, our analysis focused on the frontier molecular orbitals involved in the lumiflavin transitions associated with the first bright state (Figure 9). TD-DFT calculations, combined with natural transition orbital (NTO) analysis, showed that in the reactant, this transition is predominantly (94%) described by a single excitation from orbital 135 (energy = −6.15 eV) to the LUMO, orbital 138 (energy = −2.81 eV). In the product, the scenario changes: the transition is mainly (71%) characterized by a single excitation from orbital 134 (energy = −6.00 eV) to orbital 138, with an additional contribution (23%) from the 135 → 138 excitation. While orbital 138 (the LUMO) remains at the same energy in both reactant and product states, orbitals 134 and 135 are shifted to higher energies in the product. In particular, orbital 135 is destabilized from −6.14 to −5.98 eV, likely reflecting the reorganization of the QC region upon product formation. Moreover, while orbital 135 is strongly localized on the lumiflavin moiety in the reactant structure, in the product, both orbitals 134 and 135 become delocalized across a large portion of the network involving the bicarbonate anion (Figure 10). This delocalization reduces the energy gap between the orbitals involved in the electronic transition, leading to the observed red shift. Consistent with this interpretation, the NTO analysis of the same transition (Figure 10) shows only minimal changes in the donor and acceptor orbitals. Although excitation energies cannot be directly interpreted as simple differences between molecular orbital energies, these considerations provide a qualitative picture that highlights how relative shifts in the MO energies can rationalize the observed trends.

**Figure 10:**
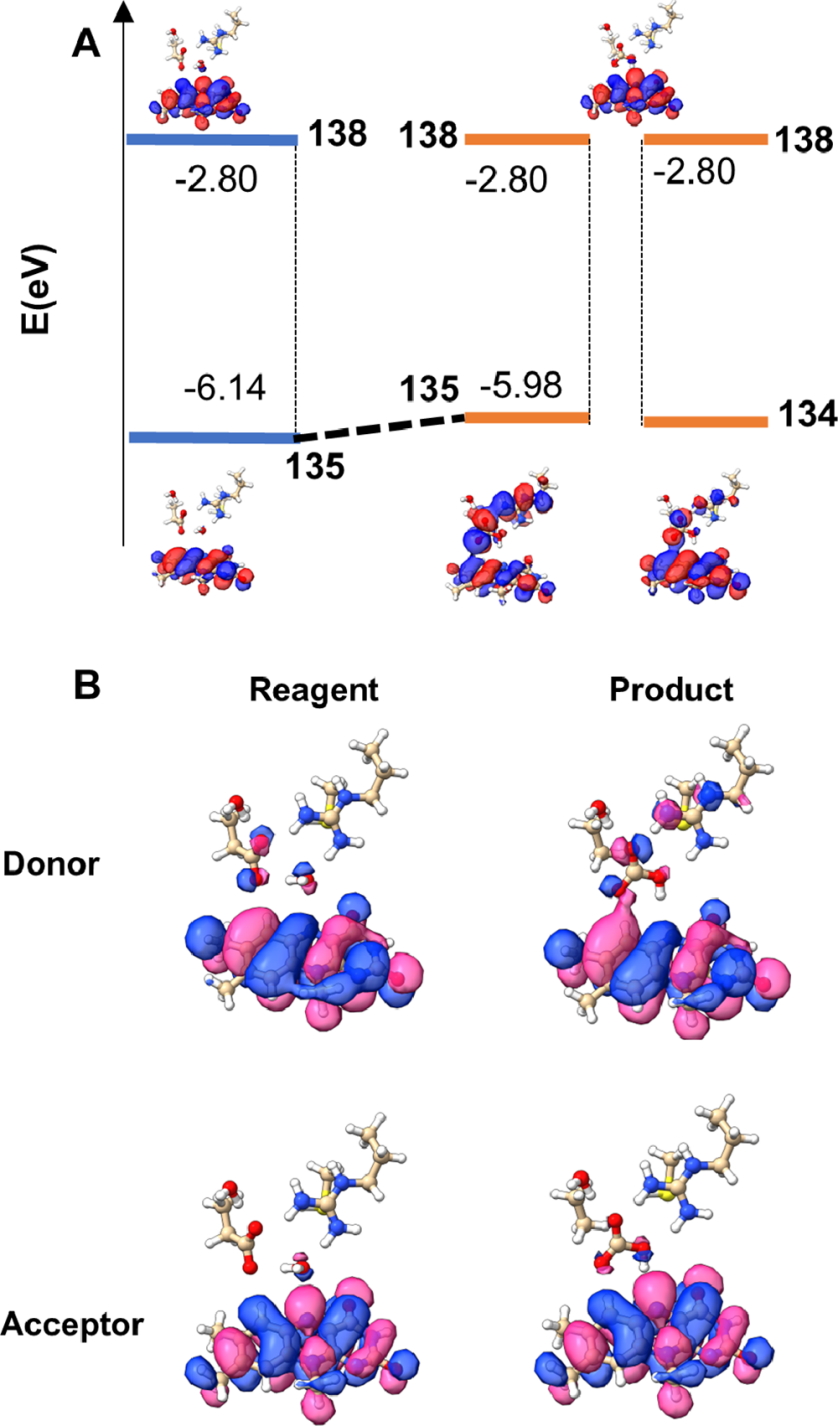
(A) Comparison between the energy gap associated with the first bright transition in the reagent and product structures. The gap is significantly reduced in the product structure due to the initially occupied orbital destabilization induced by product formation. The corresponding orbitals are reported (negative values are in blue, and positive values are in red). (B) NTOs involved in the first bright transition for reagent and product structures (negative values in blue and positive in pink).

Our calculations therefore confirm the MD-PMM results, reported in the previous section, supporting the hypothesis that the formation of *FAD*_*RS*_ is not attributable to a mere electrostatic effect but rather requires a reorganization of the active site of the molecule associated with the decarboxylation of palmitic acid and the formation of the bicarbonate anion.

## Conclusions

In this work, we have simulated the UV–vis spectrum of FAD in CvFAP using MD-PMM. The computed spectra align with experimental observations,^7,8,15^ effectively explaining the shifts in band positions. The results elucidate two principal factors that modulate the electronic properties of FAD within the CvFAP protein environment. One contributing factor is the characteristic ’butterfly’ bending of the isoalloxazine ring, which introduces alterations in the electronic structure of the flavin. Gas-phase simulations indicate that this conformational distortion predominantly affects the intensities of the absorption bands while exerting minimal influence on their positions. Instead, the pronounced red shift observed experimentally in CvFAP can be primarily attributed to the perturbation induced by the protein environment. This highlights the critical importance of incorporating environmental fluctuations through dynamic approaches such as MD-PMM in order to achieve a quantitative interpretation of experimental spectra.

Furthermore, the present results suggest that modification of the charge state of individual active-site residues, such as Cys432 and Arg451, is unlikely to produce a bathochromic shift of the magnitude observed for the FAD_RS_ species. Instead, our QM/MM calculations indicate that extensive structural rearrangements associated with product formation (including a bicarbonate anion) are more likely to be responsible for the formation of FAD_RS_. Such reorganization results in a reduced donor–acceptor orbital gap, thus explaining the experimentally observed red shift.

In conclusion, this work provides a basis for understanding the red shifts observed in different states of CvFAP. Further studies using similar hybrid approaches will be needed to clarify the reaction mechanism in detail.

## Supplementary Material



## Notes

Gas-phase TD-DFT transition energies, oscillator strengths, and dipole moments for the three lumiflavin conformations (planar, partially bent, and bent) employed in the MD-PMM calculations; comparison of M06 and B3LYP functionals in the MD-PMM calculation of lumiflavin’s UV–vis spectrum in water; spectra of the three conformational basins used for FAD’s weighted MD-PMM spectrum in CvFAP; and spectra of the three conformational basins for FAD’s weighted MD-PMM spectrum in CvFAP with altered charge topologies (Arg^0^/Cys^–1^) (PDF)
