# Supplementary material for: On the Origin of the Red-Shifted Flavin Absorption Spectra in Fatty Acid Photodecarboxylase
Source: J Phys Chem B. 2025 Dec 18;130(1):68–78. doi: 10.1021/acs.jpcb.5c05331 (PMC12794162; doi:10.1021/acs.jpcb.5c05331)
Supplement: Supplementary file 1 [file jp5c05331_si_001.pdf]

Supporting Information:
On the origin of the red-shifted flavin absorption spectra in fatty acid photodecarboxylase

Matteo Farina,[†] Gianluca Dell'Orletta,[‡] Enrico Bodo,[*,†] and Isabella Daidone[*,‡]

†*Department of Chemistry, University of Rome "La Sapienza", Piazzale Aldo Moro 5, Rome, Italy*

‡*Department of Physical and Chemical Sciences, University of L'Aquila, via Vetoio (Coppito 1), 67100 L'Aquila, Italy*

E-mail: enrico.bodo@uniroma1.it; isabella.daidone@univaq.it

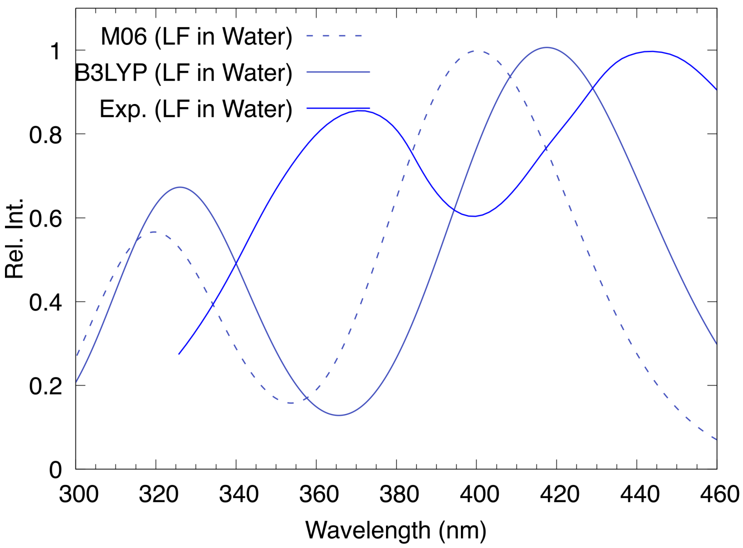

Figure S1: Comparison between the experimental absorption spectrum (thick blue line) of lumiflavin in water and the spectra calculated using the MD-Perturbed Matrix Method (PMM). Results from two DFT functionals are shown: M06 (dashed blue line) and B3LYP (thin blue line). B3LYP provides better agreement with the experimental data in terms of both peak positions and relative intensity. No scaling factor was applied in these calculations.

Table S1: Gas-phase TD-DFT transition energies, transition dipole moments and dipole moments for the lumiflavin planar structure (B1).

Trans.	E (eV)	λ (nm)	f_{osc}	D_X	D_Y	D_Z
$0 \to 1$	3.0207	410.4	0.1987	−1.5014	−0.4021	−0.5193
$0 \to 2$	3.1549	393.0	0.0003	0.0244	−0.0246	−0.0561
$0 \to 3$	3.2896	376.9	0.0003	−0.0274	0.0192	0.0449
$0 \to 4$	3.8356	323.2	0.1447	1.0975	−0.1330	0.5637
$0 \to 5$	3.9196	316.3	0.0000	0.0019	−0.0020	−0.0038
$0 \to 6$	4.0660	304.9	0.0103	0.0705	0.2991	−0.0972
$0 \to 7$	4.5964	269.7	0.0002	−0.0158	0.0151	0.0332
$0 \to 8$	4.7042	263.6	0.0834	−0.7582	0.0690	−0.3796
$0 \to 9$	4.8707	254.6	0.5862	1.7597	−0.7333	1.1305
$0 \to 10$	5.0126	247.3	0.0232	0.3956	0.0032	0.1805

State	D_X	D_Y	D_Z
0	−3.4095	−0.8903	−1.1908
1	−3.6091	−1.4871	−1.0245
2	−1.3661	−0.3504	−0.4813
3	−2.1677	−1.1352	−0.5118
4	−4.9705	−1.0095	−1.8601
5	−0.6435	−0.8873	0.0850
6	−0.6244	−0.2409	−0.1866
7	−2.8107	−1.2769	−0.7468
8	−4.1840	−1.2863	−1.3774
9	−3.2301	−1.0794	−1.0259
10	−2.6266	−1.0901	−0.7428

Table S2: Gas-phase TD-DFT transition energies, transition dipole moments and dipole moments for the partially bent (10° bending) lumiflavin structure (B3).

Trans.	E(eV)	λ(nm)	f_{osc}	D_X	D_Y	D_Z
$0 \to 1$	3.0151	411.2	0.1856	0.9979	0.2681	1.2020
$0 \to 2$	3.1527	393.3	0.0011	−0.1125	0.0139	−0.0372
$0 \to 3$	3.3038	375.3	0.0103	−0.1736	−0.0766	−0.3020
$0 \to 4$	3.8292	323.8	0.1478	0.5106	−0.1518	1.1365
$0 \to 5$	3.9091	317.2	0.0032	0.1055	0.0698	0.1302
$0 \to 6$	4.0787	304.0	0.0096	−0.1416	−0.2665	0.0672
$0 \to 7$	4.5792	270.8	0.0045	0.1214	0.0326	0.1572
$0 \to 8$	4.7239	262.5	0.0923	0.3138	−0.0763	0.8328
$0 \to 9$	4.8883	253.6	0.5534	−0.5820	0.6978	−1.9481
$0 \to 10$	4.9949	248.2	0.0105	−0.1912	0.0174	−0.2216

State	D_X	D_Y	D_Z
0	−2.2910	−0.6134	−2.8341
1	−2.6157	−1.1362	−2.7210
2	−0.8975	−0.2772	−1.2160
3	−1.7445	−0.8742	−1.5849
4	−3.0600	−0.7015	−4.1527
5	−0.7722	−0.7572	−0.4830
6	−0.3558	−0.3397	−0.5273
7	−2.2766	−0.9747	−2.3378
8	−2.7128	−0.9378	−3.1447
9	−2.3108	−0.7888	−2.6073
20	−1.9152	−0.8415	−1.9433

Table S3: Gas-phase TD-DFT transition energies, transition dipole moments and dipole moments for the bent (20° bending) lumiflavin structure (B5).

State	E(eV)	λ(nm)	f_{osc}	D_X	D_Y	D_Z
$0 \to 1$	2.9890	414.8	0.1655	1.4229	0.3409	0.3460
$0 \to 2$	3.1397	394.9	0.0006	−0.0585	−0.0100	0.0649
$0 \to 3$	3.3037	375.3	0.0225	0.4831	0.1047	0.1825
$0 \to 4$	3.7942	326.8	0.1548	−1.1662	0.1487	−0.5325
$0 \to 5$	3.9115	317.0	0.0005	−0.0529	−0.0383	−0.0266
$0 \to 6$	4.0566	305.6	0.0111	−0.0130	0.3289	−0.0592
$0 \to 7$	4.5348	273.4	0.0082	−0.2358	−0.0642	−0.1179
$0 \to 8$	4.7249	262.4	0.0835	−0.7211	0.1180	−0.4325
$0 \to 9$	4.8933	253.4	0.5058	1.7155	−0.7668	0.8295
$0 \to 10$	4.9162	252.2	0.0062	−0.0993	0.0913	−0.1829

State	D_X	D_Y	D_Z
0	−3.4089	−0.7895	−0.9205
1	−3.5340	−1.3847	−0.8667
2	−1.4718	−0.3462	−0.5239
3	−2.1652	−1.0721	−0.4234
4	−5.0105	−0.8857	−1.6017
5	−0.6043	−0.9357	−0.1799
6	−0.5323	−0.3197	−0.3856
7	−3.2333	−1.2399	−0.7721
8	−3.7007	−1.1576	−0.9756
9	−3.3026	−0.9936	−0.8231
10	−2.3657	−1.0684	−0.5006

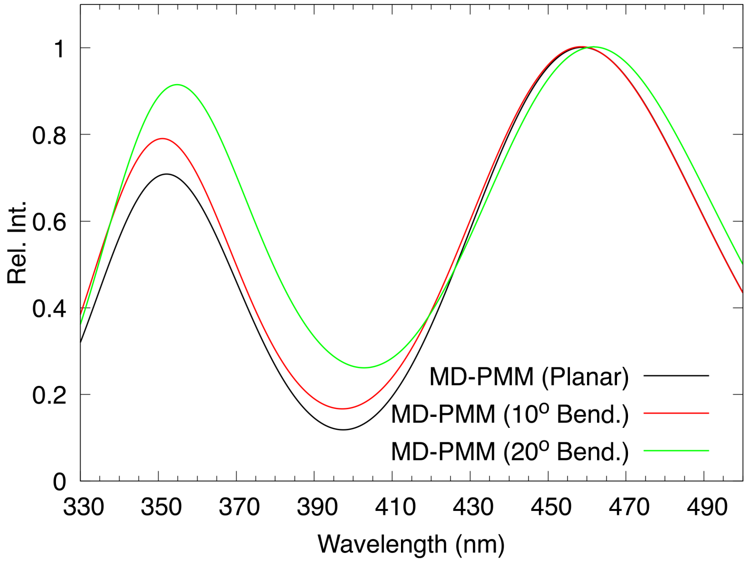

Figure S2: Absorption spectra of the three conformational basins used to compute the weighted MD-PMM spectrum of FAD in CvFAP shown in Figure 6 of the main text.

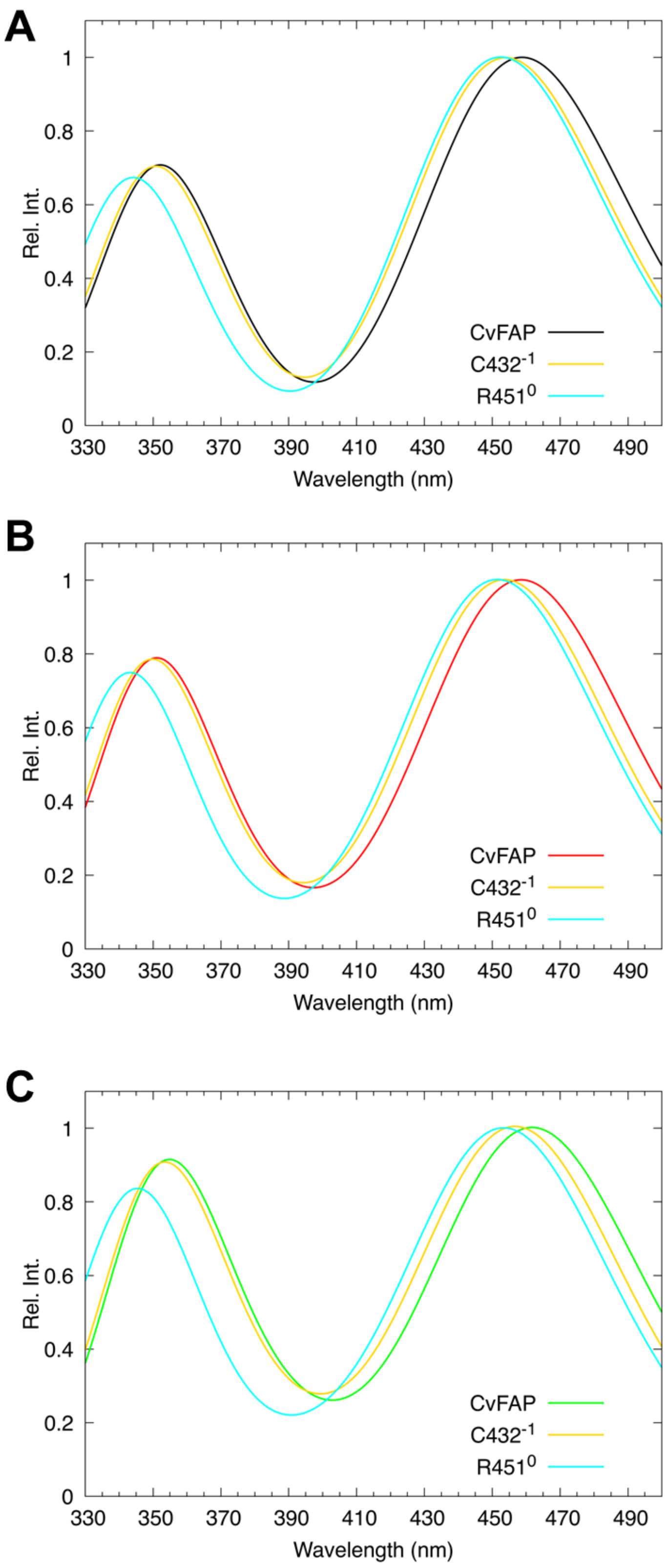

Figure S3: Absorption spectra of FAD in CvFAP obtained using MD-PMM calculations with standard Apo-state charges and with altered charges, specifically Arg451 set to neutral (0) and Cys432 to negatively charged (−1). Three lumiflavin geometries are considered: (A) planar, (B) 10° bending, and (C) 20° bending.
